# Supplementary material for: Temperature, species identity and morphological traits predict carbonate excretion and mineralogy in tropical reef fishes
Source: Nat Commun. 2023 Feb 22;14:985. doi: 10.1038/s41467-023-36617-7 (PMC9947118; doi:10.1038/s41467-023-36617-7)
Supplement: Supplementary file 1 — Supplementary Information [file 41467_2023_36617_MOESM1_ESM.pdf]

Supplementary information for:

Temperature, species identity and morphological traits predict carbonate excretion and mineralogy in tropical reef fishes

Mattia Ghilardi[1,2]*, Michael A. Salter[3], Valeriano Parravicini[4,5], Sebastian C. A. Ferse[1,2], Tim Rixen[1], Christian Wild[2], Matthias Birkicht[1], Chris T. Perry[6], Alex Berry[3], Rod W. Wilson[3], David Mouillot[5,7], Sonia Bejarano[1]

Affiliations

1. Leibniz Centre for Tropical Marine Research (ZMT), Fahrenheitstraße 6, 28359 Bremen, Germany
2. Department of Marine Ecology, Faculty of Biology and Chemistry, University of Bremen, Leobener Straße UFT, 28359 Bremen, Germany
3. Biosciences, University of Exeter, Exeter EX4 4QD, UK
4. PSL Université Paris: EPHE-UPVD-CNRS, USR3278 CRIOBE, University of Perpignan, 66860 Perpignan, France
5. Institut Universitaire de France, Paris, France
6. Geography, University of Exeter, Exeter EX4 4RJ, UK
7. MARBEC, Univer Montpellier, CNRS, Ifremer, IRD, 34095 Montpellier, France

Correspondence

*M. Ghilardi

Email: mattia.ghilardi91@gmail.com

ORCID: 0000-0001-9592-7252

Supplementary Methods

Predicting relative intestinal length

Using the largest available dataset of intestinal length of reef fishes[1], we fitted a Bayesian phylogenetic multilevel linear model to predict fish intestinal length according to individual standard length (SL) and species-level trophic level (TL) and body elongation (EL), both obtained from FishBase[2] using the R package *rfishbase*[3]. We extracted the phylogeny from the Fish Tree of Life[4] using the R package *fishtree*[5] and converted into a phylogenetic relatedness matrix[6]. We modelled the intestinal length of the ith individual in the jth species (y_{ij}) following a Student-t distribution:

$$\begin{aligned} y_{ij} &\sim \mathrm{t}(\nu, \mu_{ij}, \sigma) \\ \sigma &\sim \mathrm{t}(3, 0, 2.5) \\ \nu &\sim \Gamma(2, 0.1) \end{aligned} \tag{S1}$$

with degrees of freedom ν, scale σ, and observation specific locations μ_{ij} defined as

$$\begin{aligned} \ln(\mu_{ij}) &= \beta_{0j} + \beta_1 \ln(\mathrm{SL})_{ij} + \beta_2 (\mathrm{TL})_j + \beta_3 \ln(\mathrm{EL})_j \\ \beta_{0j} &= \gamma_0 + \gamma_{0\mathrm{phy}} \\ \gamma_{0\mathrm{phy}} &\sim \mathrm{N}(0, \tau) \\ \gamma_0 &\sim \mathrm{N}(0, 10) \\ \beta_{1:3}, \tau &\sim \mathrm{N}(0, 5) \end{aligned} \tag{S2}$$

where γ_0 is the average model intercept, $\gamma_{0\mathrm{phy}}$ is the random variation in γ_0 based on phylogenetic relatedness, and $\beta_{1:3}$ are the regression coefficients of the fixed effects. We ran the model for 4 chains, each with 4,000 iterations and a warm-up of 1,000 iterations using the R package *brms*[7].

Then, we performed a cross-validation to validate the extrapolation of intestinal length to unobserved species (i.e., species not used to train the model). No direct method to make predictions for unobserved taxa from a phylogenetic linear model (while accounting for phylogenetic relatedness) exists yet. Thus, in order to predict intestinal length for these species we followed a recent approach used to predict fish trophic guilds from a multinomial phylogenetic model[8]. We extracted posterior

draws of the phylogenetic effect ($\gamma_{0\text{phy}}$) of all species in the model and used them to estimate the phylogenetic effect of unobserved species using ancestral state reconstruction with the function *phyEstimate()* in the R package *picante*[9]. This estimation was repeated 2,000 times, each time using a different draw and randomly sampling one of 100 synthetic stochastically resolved phylogenies retrieved from the Fish Tree of Life, where species without genetic information are placed using stochastic polytomy resolution[4]. Then, we computed the intestinal length by combining, for each draw, the model intercept and slopes of the fixed effects with the predicted phylogenetic effect according to Equation (S2). For the cross-validation we used the whole training dataset (including 1,208 individuals representing 142 species and 31 families) and repeated the extrapolation approach 142 times, each time leaving out 1 species (thus simulating an unobserved species) and predicting the intestinal length for all individuals of that species. Finally, the average predictions were compared to the measured intestinal length to assess model accuracy. We observed a strong relationship between observed and predicted intestinal length ($R^2 = 0.81$ for a regression of slope 1 and intercept -0.16; Supplementary Fig. 10).

Therefore, using this model and the extrapolation procedure described above we predicted the intestinal length of all species in the carbonate dataset. Predictions were performed at the species level (not at the individual level) using a common SL for all species. The relative intestinal length (RIL) was then computed by dividing the average prediction of each species by the SL used. This procedure was necessary in order to use both RIL and body mass as potential predictors of carbonate excretion and composition, since intestinal length and body mass are strongly correlated. Since our model (Equation (S2)) does not include species-level variation on the coefficient of SL, the predicted RIL is not influenced by the SL used in the computation.

For one individual, which was identified at the genus level (*Haemulon* sp.), we predicted a genus-average RIL. First, we retrieved the genus-average TL and EL from FishBase[2]. These were then used to predict, at a fixed SL, the intestinal length of all species in the genus having genetic information in the Fish Tree of Life. Thus, each of the 2,000 posterior draws was averaged across species to obtain a full posterior distribution for the intestinal length of our unidentified species. Finally, the mean RIL was computed. This procedure was first validated using 200 randomly chosen observations of the training dataset. For each observation the species name was modified to simulate individuals identified at the genus level (the sample included 51 different genera). The intestinal lengths were then predicted and compared to the observed measurements, showing a

strong relationship ($R^2 = 0.84$ for a regression of slope 1.1 and intercept -0.44; Supplementary Fig. 11).

Supplementary Figures

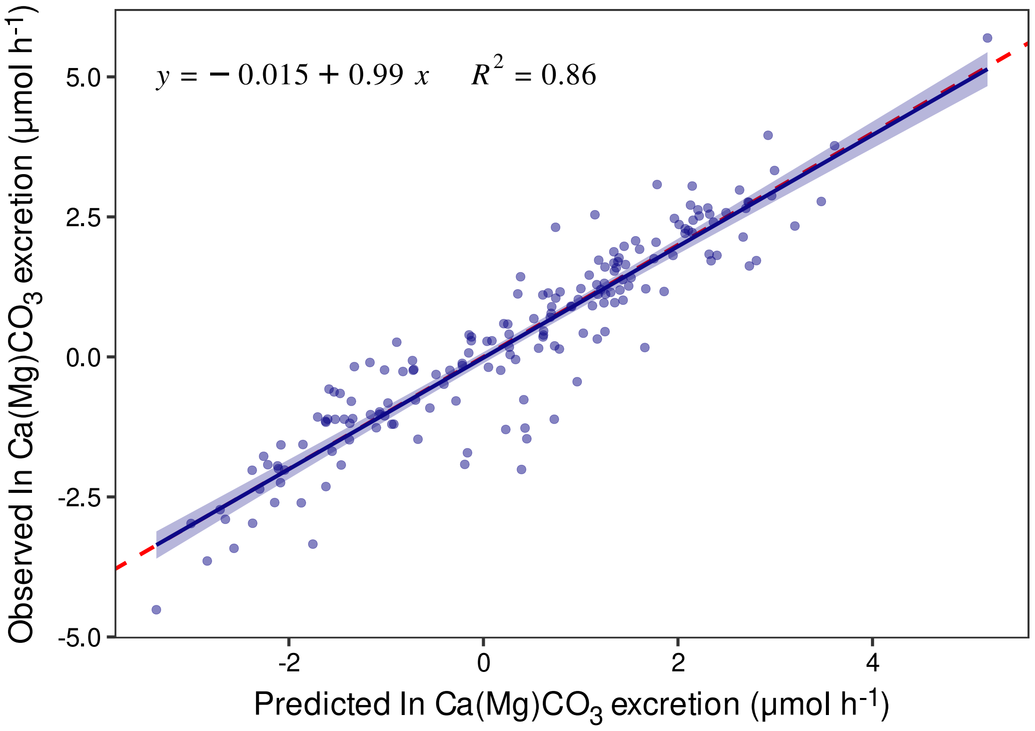

Supplementary Figure 1. Observed vs predicted total carbonate excretion rates. The solid line and ribbon show the mean estimate and 95% confidence interval, respectively, of a linear regression, whose equation and goodness of fit (R^2) are shown in the upper left of the panel. Dots represent raw data (n = 175). The dashed, red line represents the identity line (y = x). Source data are provided as a Source Data file.

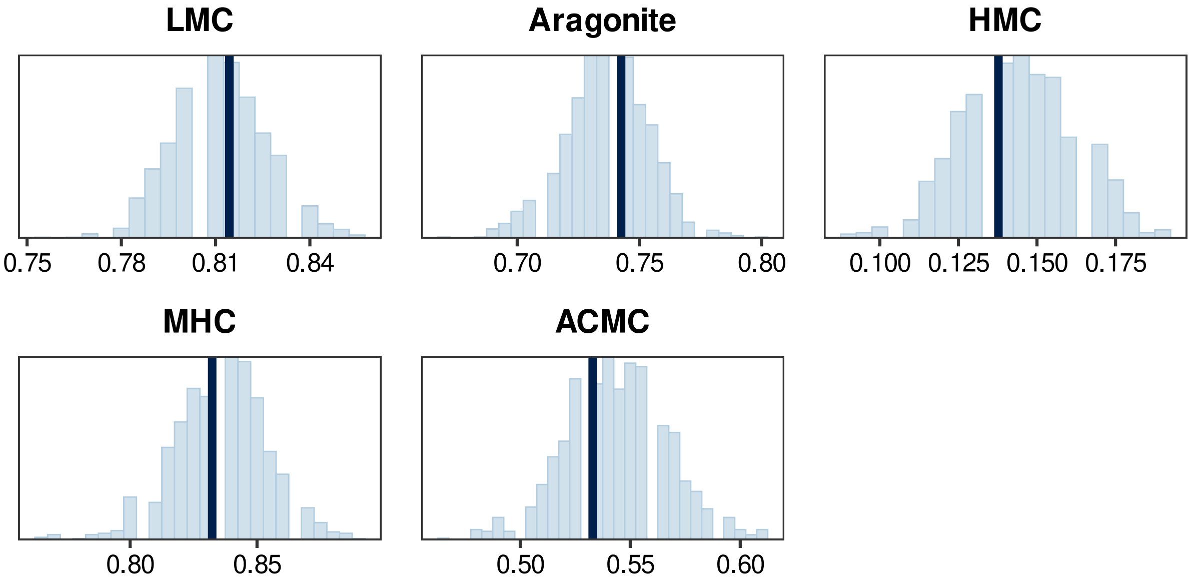

Supplementary Figure 2. Posterior predictive check of the proportion of zeros in the excretion rate of five carbonate polymorphs produced by reef fishes. Histograms represent the distribution of the proportion of zeroes in 1000 random draws of a Bayesian multivariate hurdle-lognormal model. Black lines depict the observed proportion of zeroes in the data. LMC, low-magnesium calcite; HMC, high-magnesium calcite; MHC, monohydrocalcite; ACMC, amorphous calcium magnesium carbonate. Source data are provided as a Source Data file.

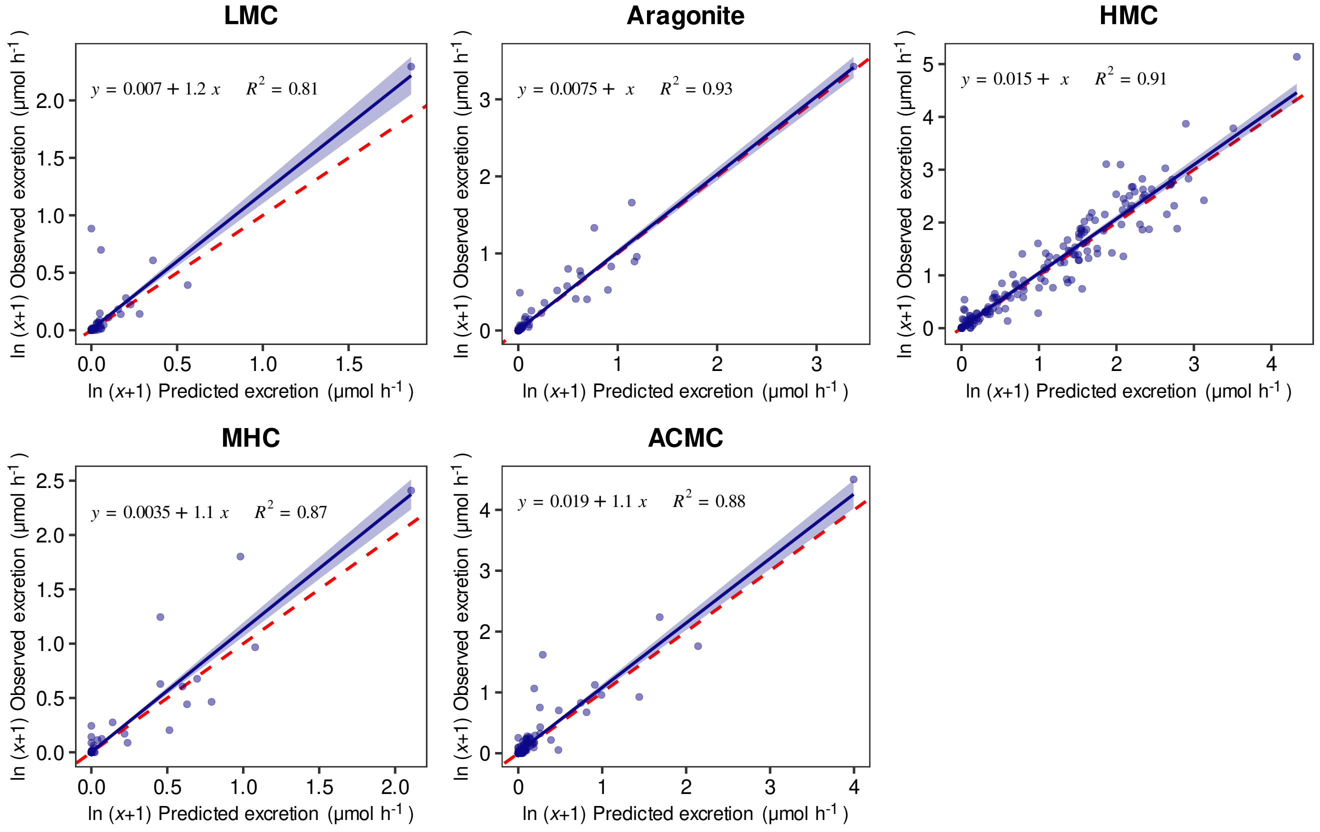

Supplementary Figure 3. Observed vs predicted excretion rates for five carbonate polymorphs produced by reef fishes. Solid lines and ribbons show the mean estimates and 95% confidence intervals, respectively, of linear regressions, whose equations and goodness of fit (R^2) are shown in the upper left of the panels. Dots represent raw data (n = 175). Dashed, red lines represent the identity lines (y = x). LMC, low-magnesium calcite; HMC, high-magnesium calcite; MHC, monohydrocalcite; ACMC, amorphous calcium magnesium carbonate. Source data are provided as a Source Data file.

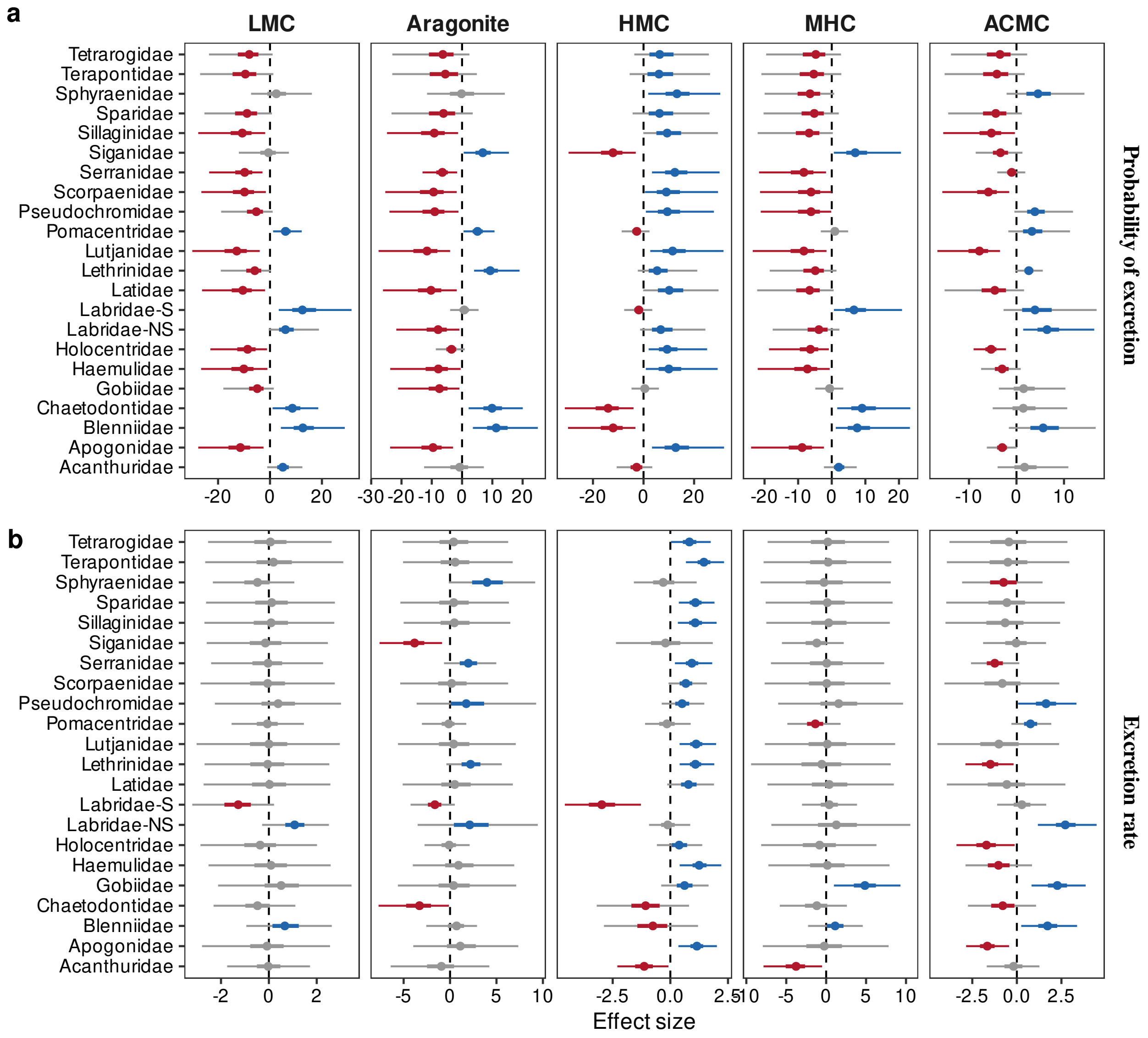

Supplementary Figure 4. (**a**) Family-specific effects on the probability of fish excreting each of five different carbonate polymorphs. (**b**) Family-specific effects on the excretion rate of five different carbonate polymorphs. Estimates are medians (circles), 50% credible intervals (CIs; thick lines) and 95% CIs (thin lines) derived from 6,000 posterior draws of a Bayesian multivariate hurdle-lognormal model. Coloured intervals indicate positive (blue) or negative (red) effects, indicating that more than 75% (if 50% CIs) or 97.5% (if 95% CIs) of the posterior density was either above or below the average model estimate, whereas grey intervals indicate that they overlap the average estimate. LMC, low-magnesium calcite; HMC, high-magnesium calcite; MHC, monohydrocalcite; ACMC, amorphous calcium magnesium carbonate; Labridae-S, scarine Labridae; Labridae-NS, non-scarine Labridae. Source data are provided as a Source Data file.

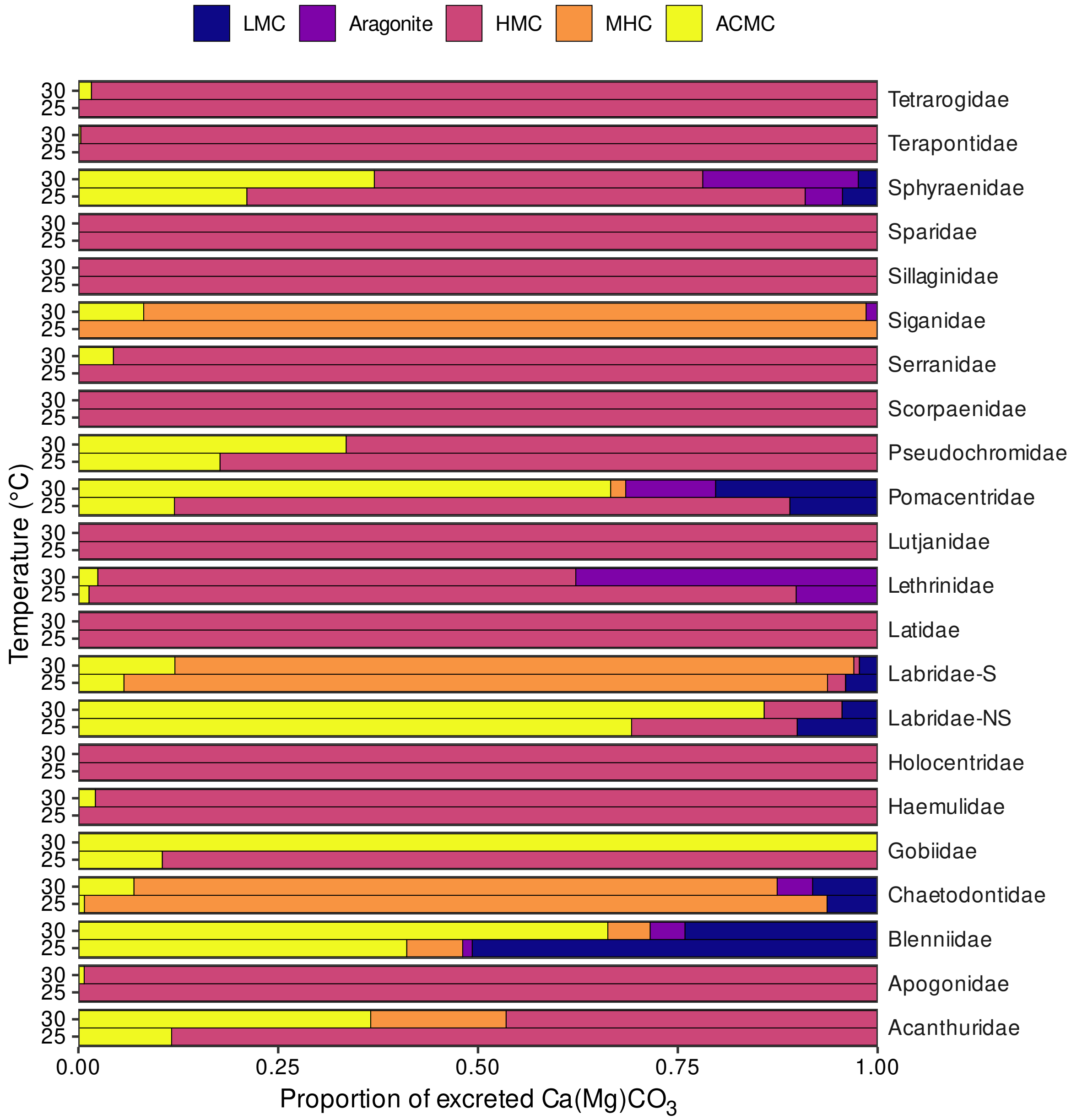

Supplementary Figure 5. Predicted average mineralogical composition of carbonates excreted by 22 fish families at two temperature levels from a Bayesian multivariate hurdle-lognormal model. Predictions are based on average family-level traits for species with genetic information in the Fish Tree of Life[4]. For each family the average biomass of an adult individual of all species was used (considering 1/2 of a species maximum length as representative of an adult individual). LMC, low-magnesium calcite; HMC, high-magnesium calcite; MHC, monohydrocalcite; ACMC, amorphous calcium magnesium carbonate; Labridae-S, scarine Labridae; Labridae-NS, non-scarine Labridae. Source data are provided as a Source Data file.

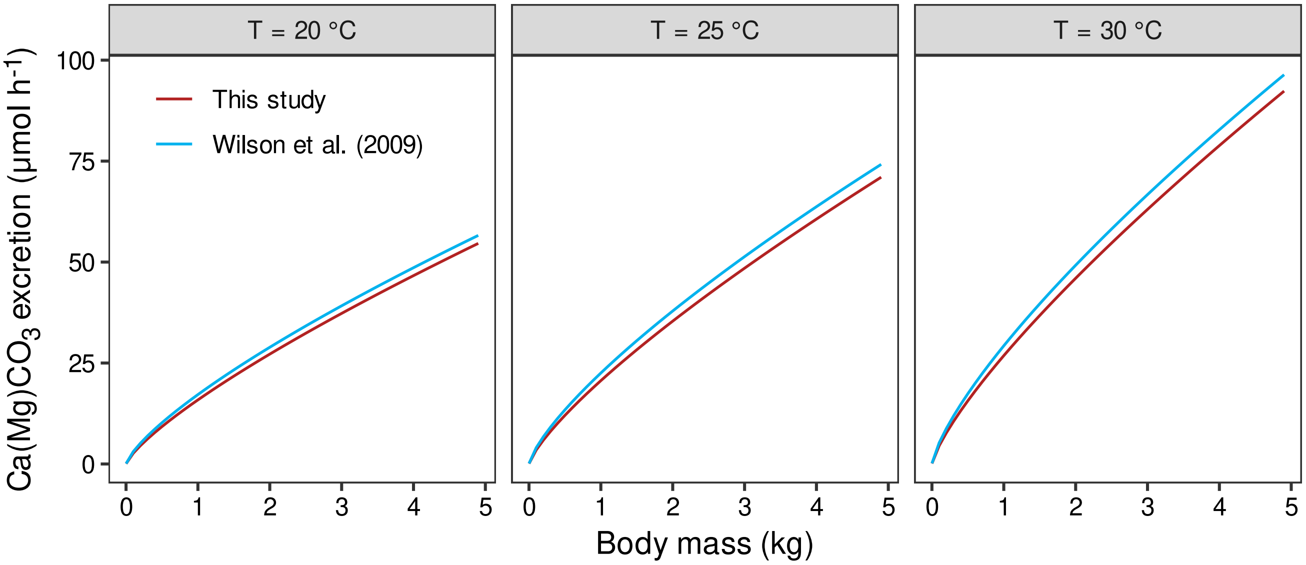

Supplementary Figure 6. Comparison of fish carbonate excretion rate predicted by the model presented in this study and that used by Wilson et al.[10] at three levels of temperature (T). Estimates of this study are average predictions, thus do not account for the effect of family, for a caudal fin aspect ratio of 1.5 and relative intestinal length of 0.5. Estimates of "Wilson's model" are predictions obtained by setting the constants ρ and α to 1. Source data are provided as a Source Data file.

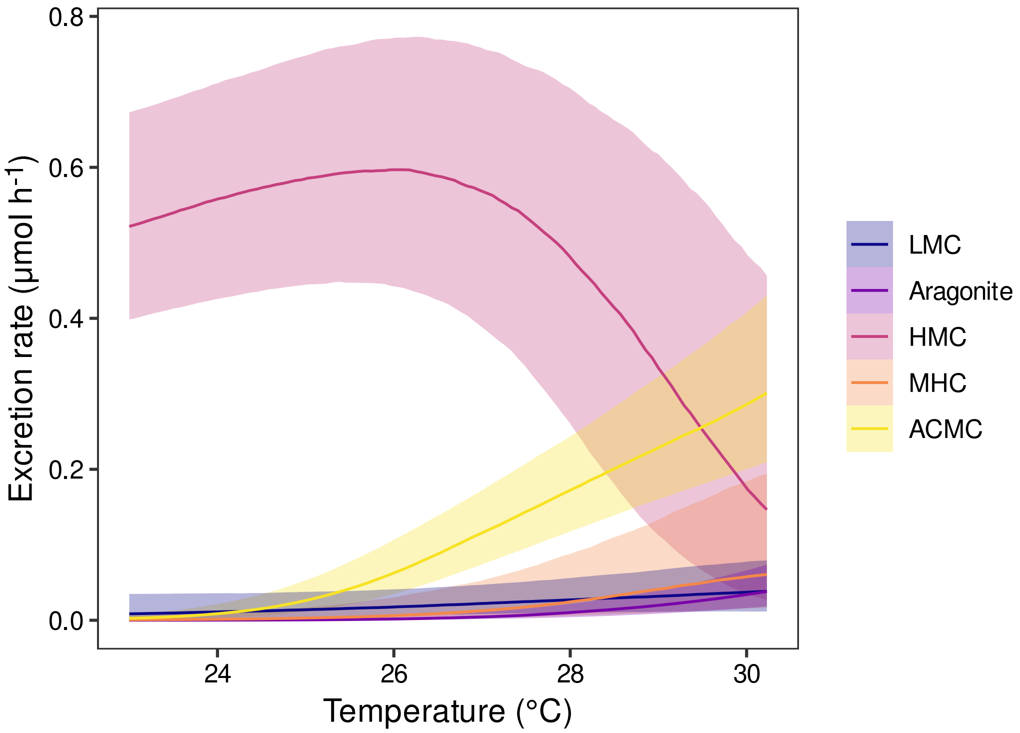

Supplementary Figure 7. Marginal effect of temperature on the excretion rate of five different carbonate polymorphs after controlling for the remaining fixed and group-level effects of a Bayesian multivariate hurdle-lognormal model by standardising the other predictors at their mean values. Coloured lines represent the median predicted fits and the ribbons show the 50% credible intervals around the estimate. LMC, low-magnesium calcite; HMC, high-magnesium calcite; MHC, monohydrocalcite; ACMC, amorphous calcium magnesium carbonate. Source data are provided as a Source Data file.

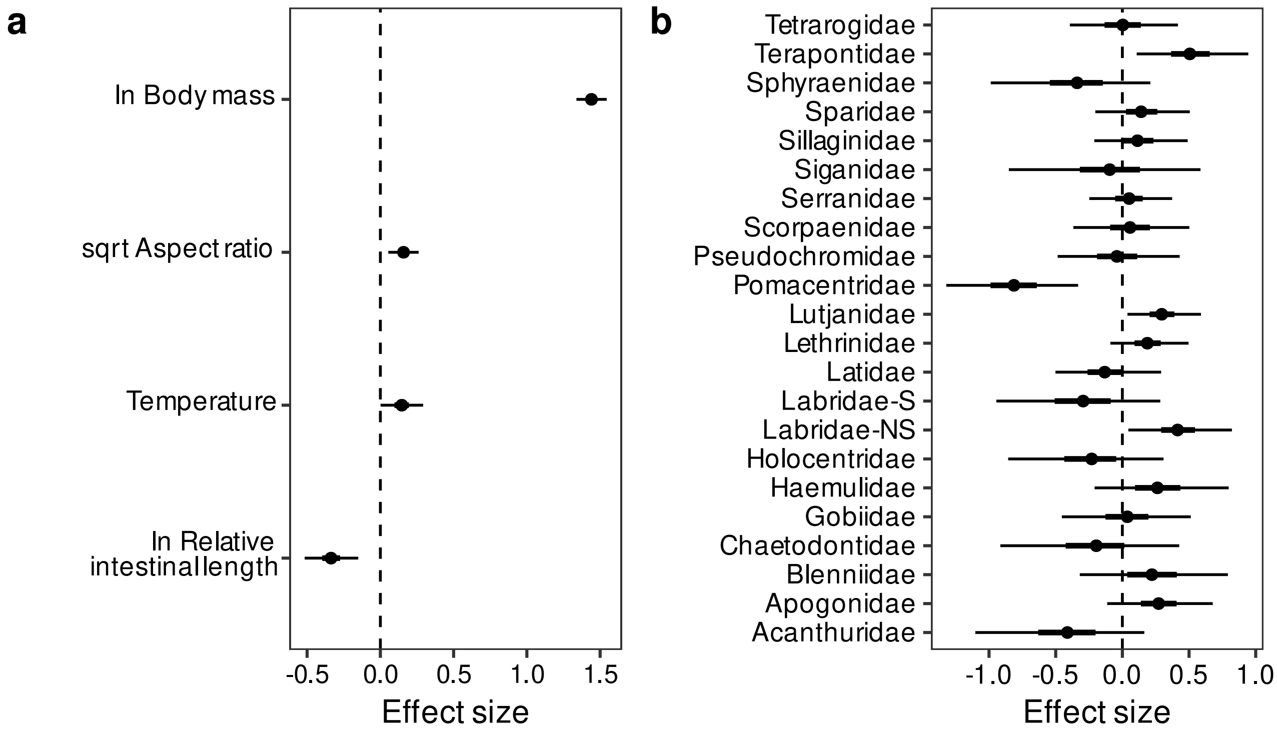

Supplementary Figure 8. Results from a Bayesian multilevel distributional regression model fitted on the corrected data (see "Carbonate excretion rates" section in the Methods). (a) Effects of fish traits and temperature on carbonate excretion rate. (b) Family-specific effects on carbonate excretion rate. Estimates are medians (circles), 50% credible intervals (CIs; thick lines; some are too narrow to be seen) and 95% CIs (thin lines) derived from 12,000 posterior draws. All predictors were standardised (mean-centred and scaled by one standard deviation) prior to fitting the model to allow for the comparison of effect sizes. Labridae-S, scarine Labridae; Labridae-NS, non-scarine Labridae. Source data are provided as a Source Data file.

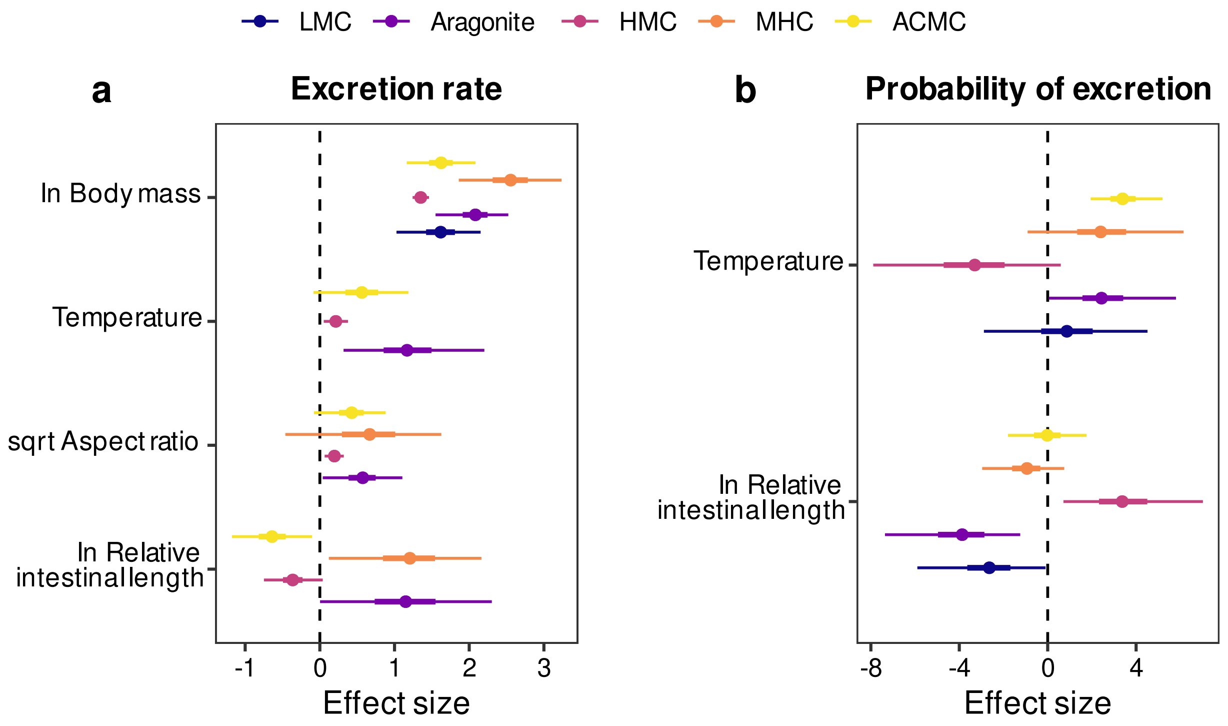

Supplementary Figure 9. Results from a Bayesian multivariate hurdle-lognormal model fitted on the corrected data (see "Carbonate excretion rates" section in the Methods). (a) Effects of fish traits and temperature on the excretion rate of five different carbonate polymorphs. (b) Effects of species' relative intestinal length (RIL) and water temperature on the probability of excreting five different carbonate polymorphs. Estimates are medians (circles), 50% credible intervals (CIs; thick lines; some are too narrow to be seen) and 95% CIs (thin lines) derived from 6,000 posterior draws. All predictors were standardised (mean-centred and scaled by one standard deviation) prior to fitting the model to allow for the comparison of effect sizes. Missing estimates correspond to effects excluded from the final model (see the "Statistical modelling" section in the Methods). LMC, low-magnesium calcite; HMC, high-magnesium calcite; MHC, monohydrocalcite; ACMC, amorphous calcium magnesium carbonate. Source data are provided as a Source Data file.

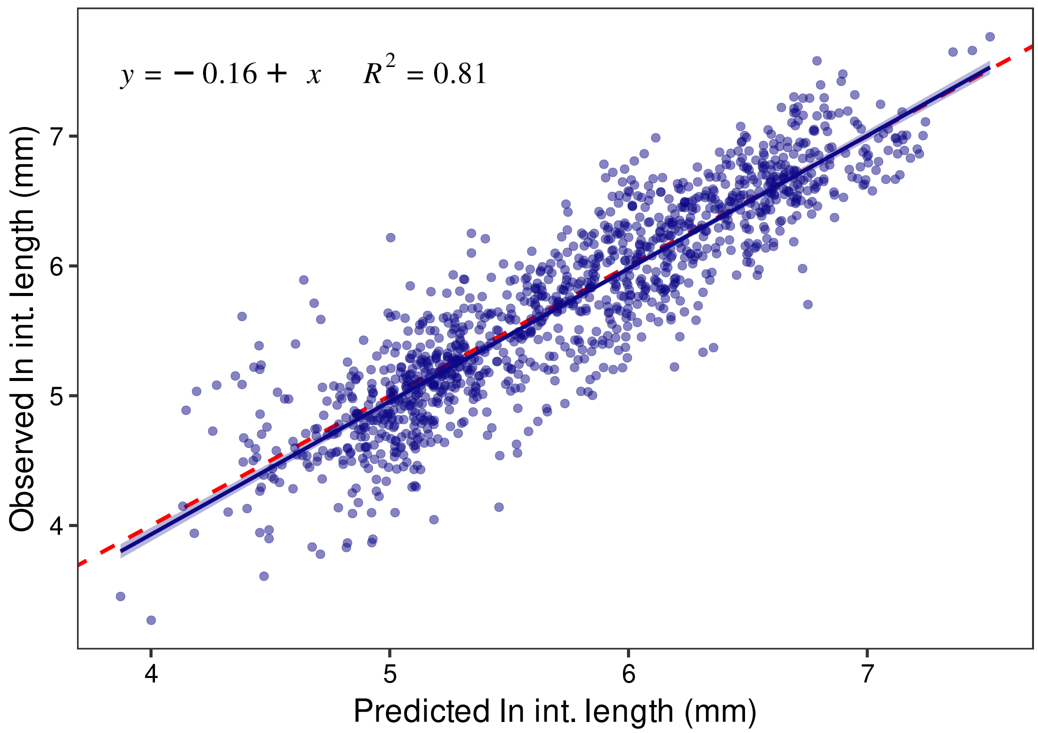

Supplementary Figure 10. Relationship between observed and predicted intestinal length for 1,208 individuals (142 species and 31 families) showing predictive accuracy for unobserved species. The solid line and ribbon show the mean estimate and 95% confidence interval, respectively, of a linear regression, whose equation and goodness of fit (R^2) are shown in the upper left of the panel. Dots represent raw data (n = 1,208). The dashed, red line represents the identity line (y = x). Source data are provided as a Source Data file.

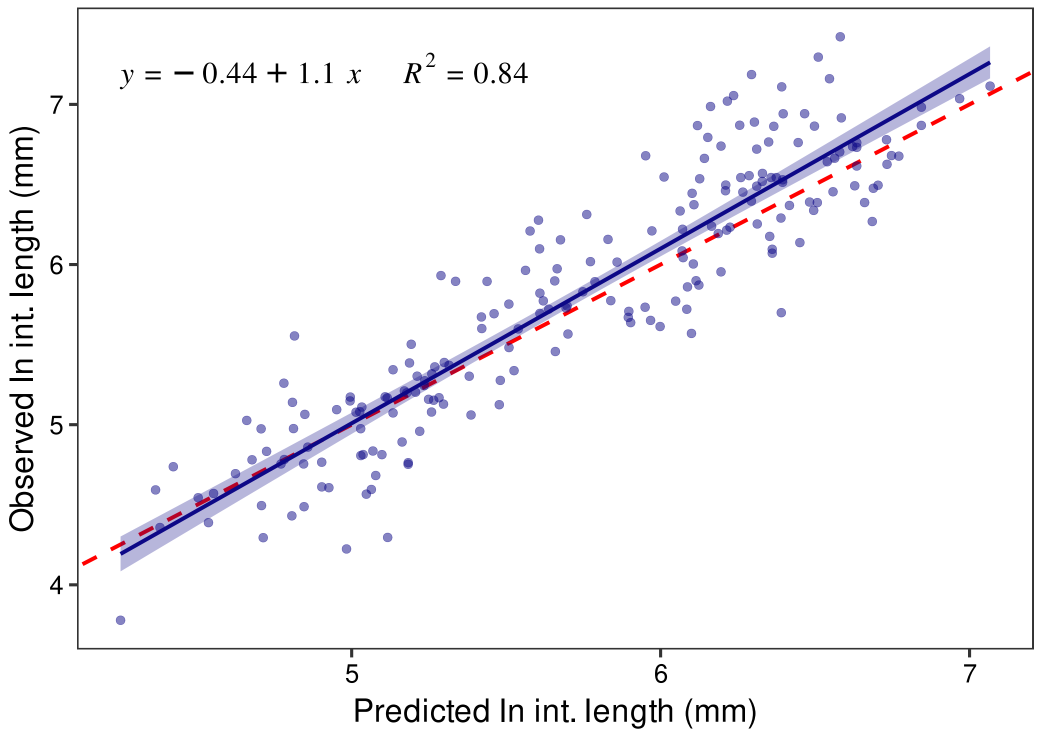

Supplementary Figure 11. Relationship between observed intestinal length and genus-level predictions for 200 individuals of the training dataset for which species names were modified to simulate unidentified species. The solid line and ribbon show the mean estimate and 95% confidence interval, respectively, of a linear regression, whose equation and goodness of fit (R^2) are shown in the upper left of the panel. Dots represent raw data ($n = 200$). The dashed, red line represents the identity line (y = x). Source data are provided as a Source Data file.

Supplementary Tables

Supplementary Table 1 The number of individuals and groups sampled per species. Included are the region, location, and family for each species. Labridae-S, scarine Labridae; Labridae-NS, non-scarine Labridae.

Region	Location	Family	Species	# of groups	# of individuals
Australia	Heron Island	Caesionidae	*Caesio cuning*	1	1
Australia	Heron Island	Labridae-NS	*Halichoeres trimaculatus*	4	6
Australia	Heron Island	Labridae-NS	*Thalassoma lunare*	1	1
Australia	Heron Island	Lethrinidae	*Gymnocranius audleyi*	3	4
Australia	Heron Island	Lethrinidae	*Lethrinus miniatus*	9	14
Australia	Heron Island	Lutjanidae	*Lutjanus adetii*	2	3
Australia	Heron Island	Lutjanidae	*Lutjanus carponotatus*	1	1
Australia	Heron Island	Pinguipedidae	*Parapercis queenslandica*	2	2
Australia	Heron Island	Pseudochromidae	*Ogilbyina queenslandiae*	2	2
Australia	Heron Island	Scorpaenidae	*Dendrochirus zebra*	1	1
Australia	Heron Island	Scorpaenidae	*Scorpaenopsis diabolus*	1	1
Australia	Heron Island	Serranidae	*Epinephelus fasciatus*	5	5
Australia	Heron Island	Serranidae	*Epinephelus quoyanus*	1	1
Australia	Heron Island	Serranidae	*Plectropomus leopardus*	3	4
Australia	Heron Island	Sillaginidae	*Sillago sihama*	5	12
Australia	Moreton Bay	Apogonidae	*Ostorhinchus limenus*	1	1
Australia	Moreton Bay	Diodontidae	*Tragulichthys jaculiferus*	1	1
Australia	Moreton Bay	Haemulidae	*Plectorhinchus picus*	1	1
Australia	Moreton Bay	Latidae	*Lates calcarifer*	12	20
Australia	Moreton Bay	Lethrinidae	*Lethrinus genivittatus*	3	16
Australia	Moreton Bay	Lethrinidae	*Lethrinus nebulosus*	3	3
Australia	Moreton Bay	Lutjanidae	*Lutjanus russellii*	11	22
Australia	Moreton Bay	Sillaginidae	*Sillago maculata*	1	4
Australia	Moreton Bay	Sparidae	*Acanthopagrus australis*	4	6
Australia	Moreton Bay	Sparidae	*Pagrus auratus*	5	8
Australia	Moreton Bay	Sparidae	*Rhabdosargus sarba*	2	5
Australia	Moreton Bay	Sygnathidae	*Hippocampus whitei*	1	1
Australia	Moreton Bay	Terapontidae	*Helotes sexlineatus*	4	29
Australia	Moreton Bay	Tetrarogidae	*Centropogon australis*	4	5
Bahamas	Cape Eleuthera	Albulidae	*Albula vulpes*	1	5

(continued)

Region	Location	Family	Species	# of groups	# of individuals
Bahamas	Cape Eleuthera	Gerreidae	*Gerres cinereus*	1	2
Bahamas	Cape Eleuthera	Haemulidae	*Haemulon sp.*	1	1
Bahamas	Cape Eleuthera	Lutjanidae	*Lutjanus apodus*	5	26
Bahamas	Cape Eleuthera	Lutjanidae	*Ocyurus chrysurus*	4	25
Bahamas	Cape Eleuthera	Mugilidae	*Mugil sp.*	1	9
Bahamas	Cape Eleuthera	Scorpaenidae	*Pterois volitans*	3	6
Bahamas	Cape Eleuthera	Serranidae	*Cephalopholis cruentata*	3	11
Bahamas	Cape Eleuthera	Serranidae	*Epinephelus guttatus*	1	1
Bahamas	Cape Eleuthera	Sphyraenidae	*Sphyraena barracuda*	4	4
Palau	Koror	Acanthuridae	*Acanthurus nigrofuscus*	2	4
Palau	Koror	Acanthuridae	*Naso annulatus*	1	1
Palau	Koror	Acanthuridae	*Naso vlamingii*	1	1
Palau	Koror	Acanthuridae	*Zebrasoma scopas*	1	1
Palau	Koror	Acanthuridae	*Zebrasoma velifer*	1	1
Palau	Koror	Apogonidae	*Ostorhinchus novemfasciatus*	3	3
Palau	Koror	Apogonidae	*Pristiapogon exostigma*	1	1
Palau	Koror	Apogonidae	*Sphaeramia nematoptera*	1	3
Palau	Koror	Apogonidae	*Sphaeramia orbicularis*	3	9
Palau	Koror	Balistidae	*Rhinecanthus verrucosus*	2	2
Palau	Koror	Blenniidae	*Atrosalarias fuscus*	2	2
Palau	Koror	Blenniidae	*Salarias ceramensis*	3	3
Palau	Koror	Blenniidae	*Salarias fasciatus*	1	1
Palau	Koror	Chaetodontidae	*Chaetodon ephippium*	2	2
Palau	Koror	Chaetodontidae	*Chaetodon lunulatus*	1	1
Palau	Koror	Chaetodontidae	*Chaetodon oxycephalus*	2	2
Palau	Koror	Gobiidae	*Amblygobius phalaena*	1	1
Palau	Koror	Gobiidae	*Amblygobius semicinctus*	3	4
Palau	Koror	Gobiidae	*Asterropteryx semipunctata*	1	5
Palau	Koror	Gobiidae	*Cryptocentrus cinctus*	1	1
Palau	Koror	Gobiidae	*Valenciennea longipinnis*	3	3
Palau	Koror	Haemulidae	*Plectorhinchus lineatus*	1	1
Palau	Koror	Holocentridae	*Myripristis adusta*	1	3
Palau	Koror	Holocentridae	*Myripristis violacea*	2	2
Palau	Koror	Holocentridae	*Sargocentron spiniferum*	2	2
Palau	Koror	Labridae-S	*Scarus dimidiatus*	2	2

(continued)

Region	Location	Family	Species	# of groups	# of individuals
Palau	Koror	Labridae-S	*Scarus globiceps*	1	1
Palau	Koror	Labridae-S	*Scarus scaber*	2	2
Palau	Koror	Lutjanidae	*Lutjanus gibbus*	1	1
Palau	Koror	Mullidae	*Parupeneus barberinus*	1	1
Palau	Koror	Muraenidae	*Gymnothorax javanicus*	1	1
Palau	Koror	Nemipteridae	*Scolopsis margaritifera*	1	1
Palau	Koror	Pomacanthidae	*Pygoplites diacanthus*	1	1
Palau	Koror	Pomacentridae	*Amblyglyphidodon curacao*	3	11
Palau	Koror	Pomacentridae	*Chromis atripectoralis*	3	16
Palau	Koror	Pomacentridae	*Dischistodus perspicillatus*	4	4
Palau	Koror	Pomacentridae	*Pomacentrus bankanensis*	1	1
Palau	Koror	Pseudochromidae	*Pseudochromis fuscus*	1	1
Palau	Koror	Pseudochromidae	*Pseudochromis marshallensis*	1	1
Palau	Koror	Serranidae	*Cephalopholis urodeta*	1	1
Palau	Koror	Serranidae	*Epinephelus merra*	2	2
Palau	Koror	Siganidae	*Siganus doliatus*	1	1
Palau	Koror	Siganidae	*Siganus puellus*	1	1
Palau	Koror	Siganidae	*Siganus punctatus*	1	1
Palau	Koror	Tetraodontidae	*Arothron nigropunctatus*	2	2
Palau	Koror	Zanclidae	*Zanclus cornutus*	1	1

Supplementary Table 2 Comparison between single end point titration and double titration in the carbonate content measured.

**Species**	**Sample ID**	**Carbonate polymorphs**	**Single titration (mmol)**	**Double titration (mmol)**	**Ratio (single/ double)**
Lethrinus atkinsoni	EMP/B-P4	HMC, ARA, ACMC	0.065	0.057	1.141
Lethrinus atkinsoni	EMP/B-P3	HMC, ARA, ACMC	0.113	0.106	1.064
Lethrinus atkinsoni	EMP/B-P2	HMC, ARA, ACMC	0.086	0.079	1.085
Lethrinus atkinsoni	EMP/B-P1	HMC, ARA, ACMC	0.054	0.049	1.108
Cephalopholis cyanostigma	CM/A-P4	HMC	0.269	0.258	1.043
Cephalopholis cyanostigma	CM/A-P3	HMC	0.168	0.157	1.069
Cephalopholis cyanostigma	CM/A-P2	HMC	0.237	0.225	1.053
Cephalopholis cyanostigma	CM/A-P1	HMC	0.212	0.201	1.059
Lutjanus fulvus	SN/C-G-P4	HMC	0.097	0.089	1.091
Lutjanus fulvus	SN/C-G-P3	HMC	0.125	0.117	1.072
Lutjanus fulvus	SN/C-G-P2	HMC	0.164	0.154	1.066
Lutjanus fulvus	SN/C-G-P1	HMC	0.138	0.129	1.068

Abbreviations:

HMC, high-magnesium calcite; ARA, aragonite; ACMC, amorphous calcium magnesium carbonate.

Supplementary Table 3 Leave-one-out (LOO) cross-validation results for 36 Bayesian regression models that examine the drivers of carbonate excretion rate. Each row represents one model, whose formula includes the fixed and random effects specified in the respective columns.

fixed	random	elpd_diff	se_diff	looic	se_looic
log(M) + log(RIL) + T + sqrt(AR)	family	0.00	0.00	485.11	36.03
log(M) + log(RIL) + T + sqrt(AR) + sqrt(ST)	family	-0.29	1.19	485.70	36.69
log(M) + log(RIL) + T	family	-1.43	2.43	487.96	35.15
log(M) + log(RIL) + T + sqrt(ST)	family	-1.99	2.64	489.09	35.89
log(M) + log(RIL) + sqrt(AR) + sqrt(ST)	family	-2.64	2.88	490.38	35.03
log(M) + log(RIL) + sqrt(AR)	family	-2.88	3.13	490.87	33.60
log(M) + log(RIL)	family	-3.14	3.93	491.38	33.36
log(M) + log(RIL) + sqrt(ST)	family	-3.35	3.79	491.82	34.65
log(M) + S + T + sqrt(AR)	family	-5.34	4.15	495.80	35.35
log(M) + S + T + sqrt(AR) + sqrt(ST)	family	-5.57	4.31	496.26	36.12
log(M) + S + sqrt(AR) + sqrt(ST)	family	-5.84	5.13	496.79	35.48
log(M) + S + sqrt(AR)	family	-6.01	5.09	497.14	34.09
log(M)	family	-6.62	5.09	498.34	33.74
log(M) + S + T	family	-7.06	4.97	499.24	34.43
log(M) + S	family	-7.27	5.59	499.65	33.59
log(M) + S + sqrt(ST)	family	-7.42	5.62	499.94	34.82
log(M) + S + T + sqrt(ST)	family	-7.84	5.09	500.79	35.18
log(M) + log(RIL) + sqrt(AR)	-	-17.50	10.06	520.11	33.78
log(M) + log(RIL)	-	-18.27	10.11	521.65	33.20
log(M) + log(RIL) + sqrt(AR) + sqrt(ST)	-	-18.44	9.94	521.99	35.14
log(M) + log(RIL) + sqrt(ST)	-	-19.53	10.01	524.16	33.92
log(M) + log(RIL) + T	-	-19.72	10.13	524.55	33.05
log(M) + log(RIL) + T + sqrt(AR)	-	-19.77	10.22	524.66	34.76
log(M) + S + sqrt(AR)	-	-20.30	10.77	525.71	34.40
log(M) + log(RIL) + T + sqrt(AR) + sqrt(ST)	-	-20.37	10.10	525.86	35.52
log(M) + S	-	-20.80	10.82	526.72	34.27
log(M) + log(RIL) + T + sqrt(ST)	-	-20.85	10.03	526.81	33.71
log(M) + S + sqrt(AR) + sqrt(ST)	-	-20.99	10.75	527.09	35.43
log(M) + S + T + sqrt(AR)	-	-21.78	10.77	528.66	34.62
log(M) + S + sqrt(ST)	-	-21.82	10.82	528.74	35.08

(continued)

fixed	random	elpd_diff	se_diff	looic	se_looic
log(M) + S + T	-	-22.05	10.82	529.20	34.21
log(M) + S + T + sqrt(AR) + sqrt(ST)	-	-22.36	10.76	529.83	35.25
log(M) + S + T + sqrt(ST)	-	-22.92	10.84	530.95	34.79
log(M)	-	-28.73	11.90	542.58	36.04
Intercept only	family	-147.32	20.77	779.75	36.10
Intercept only	-	-242.17	21.93	969.45	37.56

Abbreviations:

M, body mass; S, salinity; T, temperature; AR, caudal fin aspect ratio; RIL, relative intestinal length; ST, total sampling period; elpd_diff, difference in expected log pointwise predictive density (ELPD) between each model and the model having the largest ELPD; se_diff, standard error of the difference in ELPD; looic, LOO information criterion; se_loo, LOO standard error.

Supplementary Table 4 Leave-one-out (LOO) cross-validation results for two Bayesian multilevel regression models that examine the drivers of carbonate excretion rate. Each row represents one model, whose formula includes the fixed and random effects specified in the respective columns. The two models differ in that one also estimates the effect of the titration method on the scale parameter σ of the t-distribution (specified in the column "sigma"), while the other does not.

**fixed**	**random**	**sigma**	**elpd_diff**	**se_diff**	**looic**	**se_looic**
log(M) + log(RIL) + T + sqrt(AR)	family	method	0.00	0.00	451.27	36.70
log(M) + log(RIL) + T + sqrt(AR)	family	-	-16.92	7.61	485.11	36.03

Abbreviations:

M, body mass; RIL, relative intestinal length; T, temperature; AR, caudal fin aspect ratio; elpd_diff, difference in expected log pointwise predictive density (ELPD) between each model and the model having the largest ELPD; se_diff, standard error of the difference in ELPD; looic, LOO information criterion; se_loo, LOO standard error.

Supplementary References

1. Ghilardi, M. *et al.* Data and code of accepted version of manuscript: Phylogeny, body morphology, and trophic level shape intestinal traits in coral reef fishes (Ecology and Evolution). (2021) doi:10.5281/zenodo.5172790.

2. Froese, R. & Pauly, D. FishBase. www.fishbase.org (2021).

3. Boettiger, C., Lang, D. T. & Wainwright, P. C. Rfishbase: Exploring, manipulating and visualizing FishBase data from R. *Journal of Fish Biology* **81**, 2030–2039 (2012).

4. Rabosky, D. L. *et al.* An inverse latitudinal gradient in speciation rate for marine fishes. *Nature* **559**, 392–395 (2018).

5. Chang, J., Rabosky, D. L., Smith, S. A. & Alfaro, M. E. An r package and online resource for macroevolutionary studies using the ray-finned fish tree of life. *Methods in Ecology and Evolution* **10**, 1118–1124 (2019).

6. Hadfield, J. D. & Nakagawa, S. General quantitative genetic methods for comparative biology: Phylogenies, taxonomies and multi-trait models for continuous and categorical characters. *Journal of Evolutionary Biology* **23**, 494–508 (2010).

7. Bürkner, P. C. brms: An R package for Bayesian multilevel models using Stan. *Journal of Statistical Software* **80**, (2017).

8. Parravicini, V. *et al.* Delineating reef fish trophic guilds with global gut content data synthesis and phylogeny. *PLoS Biology* **18**, (2020).

9. Kembel, S. W. *et al.* Picante: R tools for integrating phylogenies and ecology. *Bioinformatics* **26**, 1463–1464 (2010).

10. Wilson, R. W. *et al.* Contribution of fish to the marine inorganic carbon cycle. *Science* **323**, 359–362 (2009).
