# Supplementary material for: Temperature, species identity and morphological traits predict carbonate excretion and mineralogy in tropical reef fishes
Source: Nat Commun. 2023 Feb 22;14:985. doi: 10.1038/s41467-023-36617-7 (PMC9947118; doi:10.1038/s41467-023-36617-7)
Supplement: Supplementary file 3 — Reporting Summary [file 41467_2023_36617_MOESM3_ESM.pdf]

nature portfolio

Corresponding author(s): Mattia Ghilardi

Last updated by author(s): Jan 12, 2023

Reporting Summary

Nature Portfolio wishes to improve the reproducibility of the work that we publish. This form provides structure for consistency and transparency in reporting. For further information on Nature Portfolio policies, see our Editorial Policies and the Editorial Policy Checklist.

Statistics

For all statistical analyses, confirm that the following items are present in the figure legend, table legend, main text, or Methods section.

n/a	Confirmed	
☐	☒	The exact sample size (*n*) for each experimental group/condition, given as a discrete number and unit of measurement
☐	☒	A statement on whether measurements were taken from distinct samples or whether the same sample was measured repeatedly
☐	☒	The statistical test(s) used AND whether they are one- or two-sided<br>*Only common tests should be described solely by name; describe more complex techniques in the Methods section.*
☐	☒	A description of all covariates tested
☒	☐	A description of any assumptions or corrections, such as tests of normality and adjustment for multiple comparisons
☐	☒	A full description of the statistical parameters including central tendency (e.g. means) or other basic estimates (e.g. regression coefficient) AND variation (e.g. standard deviation) or associated estimates of uncertainty (e.g. confidence intervals)
☐	☒	For null hypothesis testing, the test statistic (e.g. *F*, *t*, *r*) with confidence intervals, effect sizes, degrees of freedom and *P* value noted<br>*Give P values as exact values whenever suitable.*
☐	☒	For Bayesian analysis, information on the choice of priors and Markov chain Monte Carlo settings
☒	☐	For hierarchical and complex designs, identification of the appropriate level for tests and full reporting of outcomes
☒	☐	Estimates of effect sizes (e.g. Cohen's *d*, Pearson's *r*), indicating how they were calculated

Our web collection on statistics for biologists contains articles on many of the points above.

Software and code

Policy information about availability of computer code

Data collection	Fish trait data (caudal fin aspect ratio, trophic level, body elongation) were obtained from FishBase (www.fishbase.org) using the R package rfishbase (version 3.1.9), and the intestinal length data were obtained from the Zenodo repository (https://doi.org/10.5281/zenodo.5172790). The phylogeny used to model and predict fish intestinal length was extracted from the Fish Tree of Life (www.fishtreeoflife.org) using the R package fishtree (version 0.3.4).
Data analysis	All data analyses were conducted in R (version 4.1.3) and all models were fitted with the R package brms (version 2.15.0). All code to reproduce analyses and figures is available on GitHub (https://github.com/mattiaghilardi/FishCaCO3Model) and Zenodo (https://doi.org/10.5281/zenodo.7530092).

For manuscripts utilizing custom algorithms or software that are central to the research but not yet described in published literature, software must be made available to editors and reviewers. We strongly encourage code deposition in a community repository (e.g. GitHub). See the Nature Portfolio guidelines for submitting code & software for further information.

Data

Policy information about availability of data

All manuscripts must include a data availability statement. This statement should provide the following information, where applicable:

- Accession codes, unique identifiers, or web links for publicly available datasets
- A description of any restrictions on data availability
- For clinical datasets or third party data, please ensure that the statement adheres to our policy

The data generated and/or analysed in this study have been deposited in the Zenodo repository (https://doi.org/10.5281/zenodo.7530092). The intestinal length data used in this study are freely available in the Zenodo repository (https://doi.org/10.5281/zenodo.5172790).

Human research participants

Policy information about studies involving human research participants and Sex and Gender in Research.

Reporting on sex and gender	N/A
Population characteristics	N/A
Recruitment	N/A
Ethics oversight	N/A

Note that full information on the approval of the study protocol must also be provided in the manuscript.

Field-specific reporting

Please select the one below that is the best fit for your research. If you are not sure, read the appropriate sections before making your selection.

☐ Life sciences ☐ Behavioural & social sciences ☒ Ecological, evolutionary & environmental sciences

For a reference copy of the document with all sections, see nature.com/documents/nr-reporting-summary-flat.pdf

Ecological, evolutionary & environmental sciences study design

All studies must disclose on these points even when the disclosure is negative.

Study description	We investigated the determinants of carbonate excretion rate and mineralogical composition in reef fishes. We collected data on carbonate excretion rates from 382 individuals across 85 fish species and 35 families, and compiled trait information for each species. Then, considering only families with at least three independent observations (352 individuals from 71 species and 21 families), we used Bayesian modelling to identify the environmental factors and fish traits that predict carbonate excretion rate and mineralogy.
Research sample	The research sample consists of 382 individuals from 85 species and 35 families of reef fishes (Acanthuridae, Albulidae, Apogonidae, Balistidae, Blenniidae, Caesionidae, Chaetodontidae, Diodontidae, Gerreidae, Gobiidae, Haemulidae, Holocentridae, Labridae, Latidae, Lethrinidae, Lutjanidae, Mugilidae, Mullidae, Muraenidae, Nemipteridae, Pinguipedidae, Pomacanthidae, Pomacentridae, Pseudochromidae, Scorpaenidae, Serranidae, Siganidae, Sillaginidae, Sparidae, Sphyraenidae, Sygnathidae, Terapontidae, Tetraodontidae, Tetrarogidae, Zanclidae). A list of all species is provided in Supplementary Table 1. We focus on tropical and subtropical reef fishes as they represent most of marine vertebrate biodiversity within a small fraction of the ocean. The research sample includes some of the most abundant and biomass-rich families on coral reefs and is representative of tropical and subtropical reef fishes.
Sampling strategy	We aimed to sample as many fish families as possible (including the most abundant and biomass-rich on coral reefs), encompassing a wide range in body mass and trophic level, from a range of environmental conditions (particularly temperature). While we aimed to sample at least three individuals (or groups) per family based on the selected Bayesian modelling procedure, this was not always possible. Some observations were thus excluded from the analysis.
Data collection	At each study location fish were collected from the reef and held in tanks supplied with flow-through filtered natural seawater, except in Moreton Bay where we used filtered natural seawater in a recirculation system, and maintained at ambient conditions. To ensure that samples comprised only fish carbonate food was withheld for at least 48 h prior to sampling and during the sampling period. Carbonate pellets were collected from the tanks at regular intervals, cleaned to remove excess salts and organic material, and dried. These samples were then analysed for carbonate content, morphology, and mineralogical composition. Data were recorded on spreadsheets. MG, MB, and SB collected data from Palau. MAS, CTP, and RWW collected data from The Bahamas and Australia. AB analysed samples to compare titration methods.
Timing and spatial scale	Our data are referred to samples collected at four study locations: Eleuthera (24°50′N, 76°20′W), The Bahamas, in November 2009,

Timing and spatial scale	July 2010, May and December 2011; Heron Reef (23°27'S, 151°55'E) in Queensland, Australia, in April 2014; Moreton Bay (27°29'S, 153°24'E) in Queensland, Australia, in May 2014 and May 2015; and Koror (7°20'N, 134°28'E), Palau, in November and December 2019. Since fish continuously produce and excrete calcium carbonate, field studies could have been conducted at any time. As such, there were no strict selection criteria on the timings of data collection, other than to select times when weather conditions were expected to be reasonably stable. This was to ensure reasonable consistency of environmental parameters within research aquaria during the data collection period, and, from a practical viewpoint, to facilitate work in the marine environment.
Data exclusions	For our multilevel models with family as group-level effect, we retained only families with at least three independent observations (352 individuals from 71 species and 21 families) to reduce the risk of introducing outliers and have more robust estimates of family effects.
Reproducibility	All data and code needed to reproduce our study are available on GitHub (https://github.com/mattiaghilardi/FishCaCO3Model) and Zenodo (https://doi.org/10.5281/zenodo.7530092).
Randomization	Individual fishes were chosen to maximise the number of fish families and the range of body mass and trophic level, but collection was dictated by capture success. We controlled for the potential confounding effect of the titration method used to analyse the samples by including this variable in the model structure and performing a sensitivity analysis on method-corrected data.
Blinding	No blinding was performed as data collection was not subject to observer bias.

Did the study involve field work? ☒ Yes ☐ No

Field work, collection and transport

Field conditions	Water temperature ranged between 23 and 31 °C and salinity between 33.8 and 36.6.
Location	We collected fish carbonate samples at four study locations: Eleuthera (24°50'N, 76°20'W), The Bahamas, in November 2009, July 2010, May and December 2011, using research facilities at the Cape Eleuthera Institute; Heron Reef (23°27'S, 151°55'E) in Queensland, Australia, in April 2014, using research facilities at the Heron Island Research Station (University of Queensland); Moreton Bay (27°29'S, 153°24'E) in Queensland, Australia, in May 2014 and May 2015, using research facilities at the Moreton Bay Research Station (University of Queensland); and Koror (7°20'N, 134°28'E), Palau, in November and December 2019, using research facilities at the Palau International Coral Reef Center. At each location, fish were collected from shallow waters (0 to 15 m) typically within 1 km of the shoreline.
Access & import/export	Access, collection, and export of samples was conducted under Marine Research Permit RE-19-28 issued by the Ministry of Natural Resources, Environment, and Tourism of the Republic of Palau (10.03.2019), Marine Research/Collection Permit and Agreement 62 issued by the Koror State Government (08.10.2019), Queensland Government GBRMPA Marine Parks Permit G14/36689.1, Queensland Government DNPRSR Marine Parks Permits QS2014/MAN247 and QS2014/MAN247a, Queensland Government General Fisheries Permit 168991, Queensland Government DAFF Animal Ethics approval CA2013/11/733, approval by The Bahamas Department of Marine Resources.
Disturbance	After sampling, all fish were returned to the sites from which they were obtained, except in the event of mortalities. Mortality rate was <5% at each location, which was deemed an acceptable attrition rate within the relevant research and ethics permits.

Reporting for specific materials, systems and methods

We require information from authors about some types of materials, experimental systems and methods used in many studies. Here, indicate whether each material, system or method listed is relevant to your study. If you are not sure if a list item applies to your research, read the appropriate section before selecting a response.

Materials & experimental systems

n/a	Involved in the study	
☒	☐	Antibodies
☒	☐	Eukaryotic cell lines
☒	☐	Palaeontology and archaeology
☐	☒	Animals and other organisms
☒	☐	Clinical data
☒	☐	Dual use research of concern

Methods

n/a	Involved in the study	
☒	☐	ChIP-seq
☒	☐	Flow cytometry
☒	☐	MRI-based neuroimaging

Animals and other research organisms

Policy information about studies involving animals; ARRIVE guidelines recommended for reporting animal research, and Sex and Gender in Research

Laboratory animals	The study did not involve laboratory animals, all fishes were collected in the field.
Wild animals	For this study, fishes from 35 families (Acanthuridae, Albulidae, Apogonidae, Balistidae, Blenniidae, Caesionidae, Chaetodontidae, Diodontidae, Gerreidae, Gobiidae, Haemulidae, Holocentridae, Labridae, Latidae, Lethrinidae, Lutjanidae, Mugilidae, Mullidae, Muraenidae, Nemipteridae, Pinguipedidae, Pomacanthidae, Pomacentridae, Pseudochromidae, Scorpaenidae, Serranidae, Siganidae, Sillaginidae, Sparidae, Sphyraenidae, Sygnathidae, Terapontidae, Tetraodontidae, Tetrarogidae, Zanclidae) were collected alive using barrier nets, dip nets, clove oil or hook and line, and immediately transferred to aquaria facilities at the Cape Eleuthera Institute, Heron Island and Moreton Bay Research Stations, and the Palau International Coral Reef Center. A list of all fish species is provided in Supplementary Table 1. Fish were held in a range of tanks (60, 400, or 1400 L in the Bahamas, 10, 60, 100, 120 or 400 L in Heron Island and Moreton Bay, and 8, 80, 280, or 400 L in Palau) of suitable dimensions for different fish sizes (<1 g to 11 kg), either individually or, for particularly social species, in small groups of similar sized individuals of the same species. All tanks were supplied with flow-through locally-drawn filtered (1-5µm) natural seawater, except in Moreton Bay where we used locally-drawn filtered natural seawater in a recirculation system, and maintained at ambient conditions. After sampling, all fish were returned to the sites from which they were obtained, except in the event of mortalities. Mortality rate was <5% at each location, which was deemed an acceptable attrition rate within the relevant research and ethics permits.
Reporting on sex	Sex was not considered in this study and this information was not collected as for most species it cannot be determined externally.
Field-collected samples	A list of all fish species sampled is provided in Supplementary Table 1. Fishes were collected alive using barrier nets, dip nets, clove oil or hook and line, and immediately transferred to aquaria facilities at the Cape Eleuthera Institute, Heron Island and Moreton Bay Research Stations, and the Palau International Coral Reef Center. Fish were held in a range of tanks (60, 400, or 1400 L in the Bahamas, 10, 60, 100, 120 or 400 L in Heron Island and Moreton Bay, and 8, 80, 280, or 400 L in Palau) of suitable dimensions for different fish sizes (<1 g to 11 kg), either individually or, for particularly social species, in small groups of similar sized individuals of the same species. All tanks were supplied with flow-through locally-drawn filtered (1-5µm) natural seawater, except in Moreton Bay where we used locally-drawn filtered natural seawater in a recirculation system, and maintained at ambient conditions. After sampling, all fish were returned to the sites from which they were obtained, except in the event of mortalities. Mortality rate was <5% at each location, which was deemed an acceptable attrition rate within the relevant research and ethics permits.<br>Carbonate samples were rinsed three times with deionised water to remove saltwater and excess salts and soaked in sodium hypochlorite (commercial bleach; <4% available chlorine) for 6-12 h to disaggregate organic material. All traces of bleach were removed with further rinses with deionised water before drying the samples for 24 h at 50 °C. Dried samples were stored at room temperature for transport to UK and Germany for further analysis.
Ethics oversight	Animal collection and holding for this study was conducted in accordance with UK and Germany animal care guidelines, under approval by the Animal Care Officer of both the University of Bremen and the Leibniz Centre for Tropical Marine Research (ZMT) and under Queensland Government DAFF Animal Ethics approval CA2013/11/733.

Note that full information on the approval of the study protocol must also be provided in the manuscript.
